# Alder Distribution and Expansion Across a Tundra Hillslope: Implications for Local N Cycling

**DOI:** 10.3389/fpls.2019.01099

**Published:** 2019-10-16

**Authors:** Verity G. Salmon, Amy L. Breen, Jitendra Kumar, Mark J. Lara, Peter E. Thornton, Stan D. Wullschleger, Colleen M. Iversen

**Affiliations:** ^1^Environmental Sciences Division and Climate Change Science Institute, Oak Ridge National Laboratory, Oak Ridge, TN, United States; ^2^International Arctic Research Center, University of Alaska, Fairbanks, AK, United States; ^3^Department of Plant Biology, University of Illinois, Urbana, IL, United States; ^4^Department of Geography, University of Illinois, Urbana, IL, United States

**Keywords:** *Alnus* (alder), arctic, nitrogen cycling, nitrogen fixation, shrub encroachment, tundra, tundra greening, nutrient limitation

## Abstract

Increases in the availability of nitrogen (N) may have consequences for plant growth and nutrient cycling in N-limited tundra plant communities. We investigated the impact alder (*Alnus viridis* spp. *fruticosa*), an N-fixing deciduous shrub, has on tundra N cycling at a hillslope located on Alaska’s Seward Peninsula. We quantified N fixation using ^15^N_2_ incubations within two distinct alder communities at this site: alder shrublands located on well-drained, rocky outcroppings in the uplands and alder savannas located in water tracks along the moist toeslope of the hill. Annual N fixation rates in alder shrublands were 1.95 ± 0.68 g N m^-2^ year^-1^, leading to elevated N levels in adjacent soils and plants. Alder savannas had lower N fixation rates (0.53 ± 0.19 g N m^-2^ year^-1^), perhaps due to low phosphorus availability and poor drainage in these highly organic soil profiles underlain by permafrost. In addition to supporting higher rates of N fixation, tall-statured alder shrublands had different foliar traits than relatively short-statured alder in savannas, providing an opportunity to link N fixation to remotely-sensed variables. We were able to generate a map of the alder shrubland distribution at this site using a multi-sensor fusion approach. The change in alder shrubland distribution through time was also determined from historic aerial and satellite imagery. Analysis of historic imagery showed that the area of alder shrublands at this site has increased by 40% from 1956 to 2014. We estimate this increase in alder shrublands was associated with a 22% increase in N fixation. Our results suggest that expansion of alder shrublands has the potential to substantially alter N cycling, increase plant productivity, and redistribute C storage in upland tundra regions. An improved understanding of the consequences of N fixation within N-limited tundra plant communities will therefore be crucial for predicting the biogeochemistry of these warming ecosystems.

## Introduction

Since the late 20^th^ century, tundra regions have been greening in response to changing climate and an accelerated disturbance regime (Jia et al., 2003; Goetz et al., 2005; Lara et al., 2018). An important component of greening in the low Arctic has been the expansion of deciduous shrubs into previously graminoid-dominated tundra communities (Sturm et al., 2001; Tape et al., 2006; Myers-Smith et al., 2011). Deciduous shrubs store a greater amount of carbon (C) in live plant biomass and are associated with increased magnitude, quality, and turnover of litter nitrogen (N; DeMarco et al., 2014). Transitioning plant communities therefore have important implications for C and N cycling within tundra ecosystems that have historically been nutrient-limited (Chapin and Shaver, 1985; Hobbie, 1992; Mack et al., 2004).

As tundra plant communities in low Arctic regions shift toward greater dominance by deciduous shrubs, the trajectory of these transitioning ecosystems will likely be underpinned by keystone species like alder (Hollingsworth et al., 2010; Nossov et al., 2010). Alder broadly refers to the genus *Alnus*, which comprises approximately 25 deciduous tree and shrub species found in temperate, boreal and arctic regions of the northern hemisphere that introduce biologically available N to an ecosystem via symbiotic N fixation. *Frankia* bacteria housed within alder root nodules fix N in exchange for plant C. This exchange allows alder to colonize recently-disturbed areas with low levels of N availability such as fire scars, floodplains, deglaciated surfaces, and cryoturbated mineral soils (Crocker and Major, 1955; Uliassi and Ruess, 2002; Mitchell and Ruess, 2009; Frost et al., 2013). In addition to being an early successional species, alder is also one of the shrubs contributing to the high latitude greening trend that has been observed since the 1980’s (Goetz et al., 2005; Bhatt et al., 2010; Myers-Smith et al., 2011; Epstein et al., 2012). Ground-based observations of shrub expansion are spatially and temporally limited compared to the satellite record, but these direct observations provide important insight into the dynamics and species behind regional greening trends observed in the satellite record (Epstein et al., 2012). Shrub expansion into Alaskan tundra has been attributed in part to alder (*Alnus viridis* ssp. *crispa* (Aiton); Sturm et al., 2001; Tape et al., 2006) and recent decades have also seen elevated levels of alder recruitment at the northern boreal tree line in NW Canada (*Alnus viridis* ssp. *fruticosa* (Rupr.); Lantz et al., 2010). Warmer summer temperatures in the tundra regions of the Brooks Range and on the North Slope of Alaska have led to alder expansion along well-drained, rocky hillslopes (*A. viridis* ssp. *fruticosa*; Tape et al., 2012). Alder’s role in shrub expansion is likely greatest in Alaska and Western Canada (Myers-Smith et al., 2011), but analyses of regional satellite data generally do not distinguish alder from other arctic shrub species. The impact of symbiotic N fixation by arctic alder has therefore been difficult to assess at a landscape scale. Separating alder from other deciduous shrubs in regional analysis is valuable because alder at lower latitudes has been associated with high N inputs, low nutrient use efficiency and a relatively open, or “leaky,” N cycle (*Alnus rubra* (Bong.) in Binkley et al., 1992; *Alnus incana* ssp. *tenuifolia* (Nutt) in Clein and Schimel, 1995). In the Alps, alder expansion into N-poor grasslands has been shown to increase local soil N availability as well as the N content of neighboring plants (*Alnus viridis* (Chaix); Bühlmann et al., 2016). This caused not only a shift of N from soils into plant biomass, but also a loss of N via leaching into downslope catchments (*A. viridis*; Bühlmann et al., 2014; Bühlmann et al., 2016). The tundra biome is poor in nutrients, with N availability limiting both plant productivity and decomposition by soil microbes (Chapin and Shaver, 1985; Shaver and Chapin, 1980; Hobbie, 1992; Mack et al., 2004). Nitrogen introduced to tundra ecosystems by alders therefore has the potential to exert an important influence on the terrestrial and aquatic biogeochemistry of these rapidly warming systems.

Alder N fixation in boreal and alpine systems are relatively well-characterized (*A. incana ssp*. tenuifolia and *A. viridis ssp*. *fruticosa*; Anderson et al., 2009; Mitchell and Ruess, 2009; Hollingsworth et al., 2010; Nossov et al., 2011; Ruess et al., 2013), but we know little about tundra-dwelling alders and their impact on N cycling within the tundra biome. A quantitative investigation of N fixation by alder growing in tundra has not yet been performed despite the novelty of a N-fixing shrub expanding its range in this nutrient-poor biome. The arctic research community also lacks a way to distinguish alders from other deciduous shrubs in remotely sensed data products, something that would be invaluable for identifying areas alders may expand in the future and quantifying their impact on landscape-level N cycling. We address these knowledge gaps by examining the above- and belowground traits of *A. viridis ssp. fruticosa* growing in two distinct plant communities at a hillslope tundra site on Alaska’s Seward Peninsula. Our goal is to answer the following questions:

Q1) Does alder significantly impact local N cycling and N fixation rates in this tundra ecosystem?
Q2) What are the sources of variation in above- and belowground traits of alder, and can observed variation be used to quantify symbiotic N fixation at the landscape scale?
Q3) Is alder cover expanding at this site and if so, what are the implications for the N cycle?

We hypothesize that alder N cycling, above- and belowground traits, and range expansion will vary according to community and landscape position. Alder shrubland communities located near the crest of the hill contain alder that are tall in stature and grow in high densities. Alder savanna communities located along the toeslope of the hill contain short alder that are dispersed throughout a community of mixed shrubs and graminoids. Since large, dense stands of alder likely support more symbiotic bacteria, we hypothesize that alder in upland shrublands will fix more N, have distinct above and belowground traits, and be capable of greater expansion in response to regional warming than their counterparts in lowland savannas.

## Materials and Methods

### Study Site

The Kougarok Hillslope site (65°09'50.1"N, 164°49'34.2"W) is in the interior of the Seward Peninsula, 103 km from the town of Nome. The historic ATLAS site (65°25'46.3"N, 164°38'48.0"W; L.D. Hinzman et al., 2003) is 32 km northeast of the Kougarok Hillslope and has a mean annual air temperature of -4.02°C, growing season precipitation of 131.6 mm, and a mean snowpack depth of 82 cm (2000-2017 data, Busey unpublished data; Hinzman et al., 2003). This region of the Seward Peninsula was likely last glaciated during the early Pleistocene (Kaufman and Hopkins, 1986; Kaufman and Manley, 2004) and permafrost here is discontinuous (Hinzman et al., 2003; Schuur and Mack, 2018). Warming temperatures have impacted the Seward Peninsula in the past half century. Glacial extent in the Kigluaik Mountains to the south of the Kougarok Hillslope has been decreasing since the 1980’s, and these glaciers are expected to disappear by 2035 (Calkin et al., 1998). Remote-sensing studies suggest shrub cover is increasing across the Seward Peninsula (Silapaswan et al., 2001; Stow et al., 2004), and the white spruce forest near the village of Council is expanding into tundra regions (Lloyd et al., 2002).

**Figure 1:**
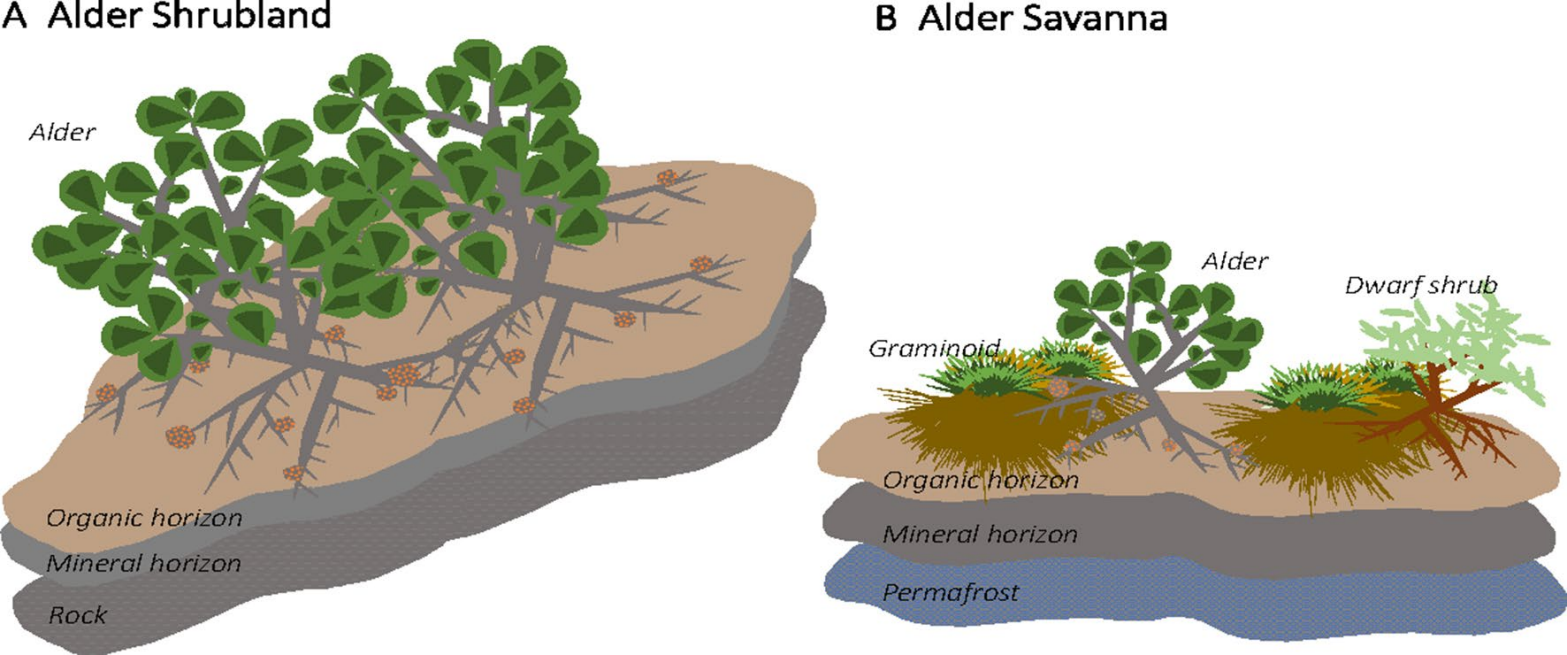
Alder (*Alnus viridis* ssp. *fruticosa*) is found in two distinct communities at the Kougarok Hillslope site. Short-statured, dispersed alder grow in lowland tundra communities (“alder savanna”) while tall alder grows in dense shrublands along the rocky ridge of the hillslope (“alder shrubland”)

The Kougarok Hillslope site encompasses an exposed, rocky outcropping surrounded by steep, well-drained slopes that transition to a lowland wetland tundra. The hillslope site spans a roughly 100-m change in elevation and a variety of tundra plant communities are present across the varying topography. *A. viridis* ssp. *fruticosa* (herein alder) grows in two of the communities present at this site: alder shrublands and alder savannas. Alder shrublands are found along the well-drained slopes below the crest of the hill and are interspersed with patches of dwarf shrub lichen tundra. Within alder shrubland communities, alder shrubs grow to height of at least 2 m and form dense, closed canopies (**Figure 1A**). Alder shrubland communities at Kougarok are similar to the upland community described in Frost et al. (2014). Soil profiles under alder shrublands at Kougarok Hillslope are quite rocky and are underlain by bedrock. Alder savannas, on the other hand, are found along the Kougarok Hillslope’s gently-sloping toeslope and are intermingled with willow-birch tundra and tussock tundra communities. Alder savannas consist of short-statured (1 m), evenly spaced alder that grow amongst other deciduous shrubs and tussock tundra along poorly-developed water tracks (**Figure 1B**) and lowland areas. Alder savanna communities at Kougarok are akin to those described in Frost et al. (2013) and the regular spacing of alder shrubs could potentially be attributed to nutrient competition, local hydrology, and/or cryogenic disturbances (Chapin et al., 1989). Within the alder savanna, alder makes up about 15% cover and the understory is characterized by sedges [*Eriophorum vaginatum* (L.) and *Carex bigelowii* (Torr. ex Schwein)], dwarf erect shrubs [*Betula nana* ssp. *exilis* (Sukaczev)*, Rhododendron tomentosum* (Harmaja)*, Vaccinium uliginosum* (L.)], lichens (*Cladonia* spp., *Cetraria* spp.) and moss [*Sphagnum* spp., *Hylocomium splendens* (Hedw.)]. Alder savanna soil profiles are underlain by permafrost and the active layer depth is approximately 40 cm. The variation in topography and presence of the same alder species in two distinct plant communities at this site makes this an ideal location for assessing the impact of alder on tundra N cycling.

### Environmental Conditions

We characterized soil pH, organic horizon depth, soil temperature at 2 cm depth and soil moisture at 8 cm depth within these two alder communities. Surface soil moisture and temperature sensors were installed in July 2016 (2 per community; H21-002, S-TMB-M002,S-MBD-005, Onset Computer Corporation, Bourne, MA). Data from July 2016 through July 2018 were summarized for summer (June-August) versus winter (September-May) seasons as well as on an annual basis. In 2016, we collected four replicate soil cores from alder shrubland and alder savanna communities. The depth of organic horizon was recorded in the field, and pH of surface soils was measured in the laboratory. In early September of 2017, we measured soil profile depth at five replicate patches of alder shrubland and alder savanna using a thaw probe. Environmental data and detailed methods are publicly available (see https://doi.org/10.5440/1346195 and https://doi.org/10.5440/1346200). 

### Soil Nutrient Availability and Depth

Soil inorganic N and phosphorus (P) availability were measured using anion- and cation-binding resins deployed at 30 locations across the Kougarok Hillslope. There are a total of six plant communities present at Kougarok Hillslope, with alder shrublands and alder savannas representing just two of the six. In 2016, resin plots were established across the six plant communities (n = 5 replicates per community) and a 10-cm deep PVC resin-access tube was installed (WECSA, LLC, Saint Ignatius, MT, USA). Resins were deployed for the summer and winter seasons from July 2016 through September 2017. In the lab, resins were rinsed with distilled water, air-dried, and serially extracted three times with 20 ml 2 M KCl solution. The three batches of extract were combined, filtered, and frozen prior to analysis of NH_4_, NO_3_, and PO_4_ on a Lachat autoanalyzer (Lachat QuikChem 8500, Hach Company, Loveland, CO). Concentrations were blank-corrected and standardized to µg N or P extracted per unit resin surface area. Resin-N was calculated as the sum of extracted NH_4_-N and NO_3_-N. Resin data and detailed methods are publicly available (see https://doi.org/10.5440/1346201). 

### Aboveground Plant Traits

We measured a suite of aboveground traits on alder growing in alder shrubland and alder savanna communities. We recorded the height individual alder shrubs and measured the basal diameter of all ramets associated with the shrub at the soil surface. To determine whether ramets were part of the same alder individual, ramets were shaken and shallow, coarse roots were traced. For each alder individual we collected 10 sun and 10 shade leaves. For the purposes of this study, we defined shade leaves as those present in the lower canopy where there was the potential for shading by upper canopy leaves, neighboring shrubs, or tussock mounds. Specific leaf area (SLA, cm^2^ leaf area g^-1^ dry leaf) was determined from leaf punches of known diameter that were counted, pooled, dried, and weighed prior to being ground for elemental and isotopic analysis. Leaf %C was measured on an elemental analyzer (Costech) while leaf %N and δ^15^N were measured using isotope ratio mass spectrometry (IRMS, Integra CN, SerCon, Crewe, UK). Leaf %P was measured by Kjeldahl digest and resulting solutions were run on a Lachat QuikChem 8500 analyzer (Lachat Instruments, Loveland, CO, USA). SLA and leaf %N and leaf %P were used to calculate N_area_ (g N m^-2^) and P_area_ (g P m^-2^). In September 2017, senesced alder litter was collected from alder shrublands by brushing a hand along branches. Litter was not collected from alder savanna communities. SLA of litter assumed a 7% loss of dry mass during senescence and resorption efficiency (%) during senescence was calculated as the change in N_area_ and P_area_ (Norby et al., 2000; Uliassi and Ruess, 2002).

Leaf samples from non-alder species were collected in July 2016 at 12 resin plots. Plots were chosen so that all six plant communities at Kougarok Hillslope were represented (n = 2 replicates per community). Foliar chemistry and SLA were measured as described above. The leaf trait data associated with alder and non-alder samples are publicly available (https://doi.org/10.5440/1346199). 

### Alder Cover Using Remotely-Sensed Data

Remote-sensing datasets from multiple sensor platforms including EO-1 Hyperion, SPOT-5, Landsat and the USGS IfSAR-based digital elevation model (**Supplementary Table 1**) were compiled using a multi-sensor fusion approach similar to that employed by Langford et al. (2019). The spectral properties of alder shrubland were particularly distinct within data from the EO-1 Hyperion hyperspectral instrument (**Supplementary Figure 1**). All remote-sensing datasets were processed at 5-m spatial resolution for analysis. A k-means, clustering based, unsupervised classification was then performed on the 211-dimensional remote sensing dataset. This resulted in classification of the entire Kougarok Hillslope into 50 clusters with similar spectral properties that were compared to ground-based observations of alder shrubland at Kougarok Hillslope (https://doi.org/10.5440/1465967). Two of the clusters were found to capture the spectral characteristics of alder shrublands and were subsequently used to generate a spatial map of alder shrubland distribution. The unsupervised classification-based (UCB) alder shrubland map was validated further by comparison with GPS coordinates of alder shrublands recorded during field sampling (red Xs in **Figure 4A**).

A time series of four cloud-free, high-resolution aerial and satellite images was analyzed using established photo-interpretation methods to determine whether alder shrubland distribution has changed in the last half-century (Lara et al., 2018; Frost et al., 2014). Geospatial image products included: 1) a 1956 panchromatic aerial photograph from USGS, 2) a 1985 color-infrared aerial photograph from the Alaska High Altitude Aerial Photography, 3) a 2006 panchromatic OrbView-3 satellite image, and 4) a 2014 multispectral Worldview-2 satellite image. All images were acquired during mid- to late summer and orthorectified, georeferenced (mean RMS error: 2.5 m), and resampled to 1 m resolution. Alder shrubland patches were digitized manually in each image at a map scale of 1:2,500 in ArcMap GIS (ArcMap 10.6; ESRI, Redlands, CA, USA). This time-series was only able to characterize change in alder shrublands; alder within alder savanna communities do not form homogenous canopies larger than 1 m in diameter and were therefore below the detection limit of the image products.

### Nodule Biomass

Nodule biomass per unit ground area was determined by extensive collection of shallow soil cores during June and July of 2017, similar to methodologies described in Ruess et al. (2013) and Uliassi and Ruess (2002). Cores were taken to a depth of 15 cm or until rock, large-diameter coarse roots, or standing water were encountered. All cores were collected with a power drill connected to a 7.3-cm diameter circular hole-saw. Measuring nodule biomass in these two plant communities required different sampling techniques due to the fact that alder in shrubland communities formed a closed canopy while alder in savanna communities grew interspersed with sedges and a variety of other low-statured evergreen and deciduous shrub species.

In five distinct stands of alder shrubland, replicate 2.5 × 2.5 m square plots were established and a grid containing 64 cells was demarcated. One soil core was collected per grid cell unless soil surface was inaccessible due to the presence of by rock or alder stem. We recorded which grid cells had individual alders rooted in them. Nodule biomass plots in the alder shrubland community contained between 4 and 8 individual alder shrubs. This sampling protocol resulted in destructive sampling of 3.9% of the total plot area and involved the collection of 288 soil cores across the five plots.

In alder savanna communities, alder shrubs were evenly dispersed with approximately one shrub per 4.1 m^2^ (unpublished data). A sampling method like the one utilized in the alder shrublands would therefore oversample the space between alder shrubs and under sample the areas closest to the shrub where coarse root biomass is highest. In order to take the spatial structure of the alder savanna community into account, we decided to sample nodule biomass within 10 circular plots, each with an the alder at the center. An inner circle within the plot spanned the canopy of the alder, while an outer circle extended this radius by 50 cm. Each plot contained only one alder shrub, located at the “bullseye” of the circles. On average, nodule plots in the alder savanna were 3.75 m^2^ (±0.47). The concentric circles were divided into eight equal slices and we collected approximately twice as many cores from the inner circle as from the outer circle. This sampling protocol resulted in destructive sampling of 3.4% of the total plot area and involved the collection of 299 soil cores across the 10 plots. Following collection, all soil cores were frozen and shipped to Oak Ridge National Laboratory (ORNL) for processing.

In the laboratory, thawed cores were examined for 10 min and live nodules were collected. Live nodules were differentiated from dead nodules based on their lighter color.and structural integrity (Ruess et al., 2009). Dead nodules were dark, spongy, and easily pulled apart. The core was sorted for additional 5 min intervals until an interval passed without discovery of live nodules. Live nodules were then rinsed, dried, and weighed. No discernable spatial patterns were observed; nodule biomass per soil core did not increase with proximity to alder stems in alder shrublands or in alder savannas. There was similarly no significant relationship between shrub basal area (average cm^2^ per shrub or shrub cm^2^ per m^2^) and nodule biomass within the data from either community. This result differed from that of Ruess et al. (2009) who used shrub basal area to scale nodule biomass measurements within stands of boreal alders. Given absence of a spatial pattern in nodule biomass observations and absence of community-specific relationships between basal area and nodule biomass, we decided to calculate average nodule biomass values for the two communities directly from the nodule biomass plots rather than scaling based on correlation with a covariate. Within alder shrublands, nodule biomass per m^2^ ground area was averaged across all soil cores collected in the square gridded plots (n = 5 plots). In alder savannas, nodule biomass was calculated as the area-weighted average of values from inner- and outer circles (n = 10 plots). Since alder savanna nodule biomass plots were similar in size to the overall density of shrubs in the alder savanna community, we feel that this approach adequately takes into account the spacing of alders within the alder savanna community. Nodule biomass data and detailed methods are publicly available (see https://doi.org/10.5440/1493669).

### N Fixation Within Nodules

Rates of N fixation within alder root nodules were determined in the field by incubating excised nodules with ^15^N-labeled N_2_ gas, as described in Anderson et al. (2004). Rates of N fixation were determined for 21 alder shrubs growing in shrublands and 12 alder shrubs growing in alder savannas in July of 2017 and 2018. Briefly, ∼2.5 g fresh weight of nodules was collected and placed immediately in a 60-ml syringe with 10 ml of 98 atm% ^15^N_2_ gas. A subsample of headspace gas was collected. After 10 min, the incubation was halted by flash-freezing the nodules in liquid N. Another ∼2.5 g of fresh nodule biomass was collected from the same alder individual to determine the initial (natural abundance) ^15^N signature of the incubated nodules. Nodule ^15^N and %N were determined by isotope ratio mass spectrometry (IRMS, Integra CN, SerCon, Crewe, UK) at ORNL. Headspace gas samples were sent to UC Davis Stable Isotope Facility for determination of atm% ^15^N_2_ (^15^N_headspace_). Atom percent excess of the nodule (APE_nodule_) was calculated as the difference between atm% ^15^N of incubated and non-incubated nodules. The rate of N fixation (Nfix) in µmole N g^-1^ nodule h^-1^ was calculated as:

Nfix=(APEnodule×Nnodule)incubationtime×N15

where N_nodule_ was the µmole N present per g dry nodule. Rates of N fixation within alder nodules and detailed methods are publicly available (see https://doi.org/10.5440/1493688). 

### Scaling N Fixation Inputs

Peak growing season N fixation was calculated as the product of nodule biomass (g nodule m^-2^ ground) and N fixation (µmole N g nodule^-1^ h^-1^). Maximal daily N fixation measurements (µmole N m^-2^ ground day^-1^) were then scaled to the entire growing season using a step function that assumes daily rates are half-maximal from 20 to 31 May, maximal from June 1 to August 15, half-maximal for the last 2 weeks of August, and quarter-maximal for the first 2 weeks of September (Uliassi and Ruess, 2002; Anderson et al., 2004; Ruess et al., 2009; Ruess et al., 2013). This assumes that daily N fixation at peak growing season is stable throughout the day, as has been observed in *A. incana* ssp*. tenuifolia* (Huss-Danell et al., 1992).

### Statistical Analyses

All statistical analyses were performed in R (R Core Team, 2018). Differences in environmental observations and aboveground alder traits of alder shrubland and alder savanna communities were compared using t-tests (p < 0.05). The effect of deployment date and plant community on resin-available resin-N and resin-P were initially determined with a two-way ANOVA. Plant community provided little explanatory power for the variation in resin-N observed, so we explored the relationship between resin-N availability and the distance to the nearest alder shrubland using the spatial data analysis packages *ggmap* (Kahle and Wickham, 2013), *sp* (Pebesma and Bivand, 2005), *raster* (Hijmans, 2019), *rgeos* (Bivand and Rundel, 2018) and *rgdal* (Bivand et al., 2018). The minimum 2-dimensional distance between each resin-N measurement and the nearest edge of the alder shrubland classification region in the modern, UCB map was determined and then the elevational difference between points was calculated. For resin-N measurements taken within the mapped extent of alder shrubland, the distance and elevation difference measures were both considered zero. The relationship between resin-N availability and the distance to alder shrubland (d) was fit to a negative exponential model using generalized non-linear least squares in the *nlme* package.

$$TIN=Ae^{-bd}$$

This two-term model fit was compared to models in which A and b were fitted to summer and winter deployments separately (A_winter_, b_winter_, & A_summer_, b_summer_) as well as a model fit to a common extinction coefficient (b) and deployment-specific intercepts. Nested models were compared based on a likelihood ratio test, and non-nested models were compared by AIC. The 95% confidence intervals for the final model fit were determined by bootstrapping (1,000 iterations).

A multiple linear regression model was used to examine the relationship between foliar chemistry of non-alder species and winter resin-N. Alder data were excluded from this analysis because the goal was to determine whether alder N inputs were impacting nearby species %N in addition to impacting resin-N. Leaf %N and δ^15^N of non-alder species were averaged for the plant functional types (PFTs) present in each plot. The relationship between nodule biomass and sun leaf N:P was analyzed with a similar simple multiple linear regression model.

The covariance of alder traits was also explored using Principal Components Analysis (PCA). PCAs were performed on the covariance matrix of aboveground traits (height, sun leaf SLA, sun leaf %N, sun leaf δ^15^N, sun leaf %P) and either nodule biomass or rates of N fixation. Following PCA analysis, the significance effect of traits on principal components was determined by bootstrapping eigenvectors (*p* < 0.05).

## Results

### Environmental Conditions

Soils within alder shrubland communities had much thinner organic horizons than alder savannas (**Table 1**). These two plant communities both had slightly acidic surface soils, and shallow soil temperatures were similar when compared on an annual and a seasonal basis. During the winter months (September through May), surface soils in alder shrublands were wetter than surface soils of alder savanna communities, potentially due to the accumulation of drifting snow around the tall alder shrubs that forms a deeper snowpack than at the toeslope. The wetter winter season in alder shrublands drove a significant difference in annual surface soil moisture content between the two communities. In deeper portions of the soil profile, however, it is likely that alder savannas had a greater moisture content; soils at 15-cm depth in inter-tussock areas of alder savannas were often saturated (*personal observation*). Indeed, soil moisture measured on 15 cm deep bulk soil in July 2017 was significantly greater alder savannas than alder shrublands (51.1% versus 30.4%, p = 0.01; McCaully et al, in preparation).

**Table 1:** Annual average surface soil temperature and soil volumetric water content within plant communities containing alder.

Soil Properties			
	Surface soil pH	Depth of organic horizon (cm)	Soil Profile Depth (cm)
Alder Shrubland	4.89 ± 0.09	7.13 ± 1.48 *	26.86 ± 2.55 *
Alder Savanna	4.69 ± 0.14	21.25 ± 2.69 *	38.96 ± 1.78 *
	*Soil Temperature (C°)*		
	**Annual**	**Summer**	**Winter**
Alder Shrubland	1.24 (-13.89, 14.96)	8.80 (2.34, 14.96)	-0.36 (-13.89, 11.95)
Alder Savanna	0.73 (-14.16, 20.48)	8.89 (0.47, 20.48)	-1.00 (-14.16, 15.44)
	*Soil VWC (m* *^3^* */m* *^3^* *)*		
	**Annual**	**Summer**	**Winter**
Alder Shrubland	0.26 (0.11, 0.41) *	0.30 (0.21, 0.37)	0.25 (0.11, 0.45) *
Alder Savanna	0.19 (0.00, 0.48) *	0.30 (0.17, 0.41)	0.16 (0, 0.49) *

Data is from summer 2016 through summer 2018 and summer was defined as June, July & August. Averages are followed by minimum and maximum values in parentheses or ± standard error. Asterisk (*) and bold indicate statistically significant differences between communities (p < 0.05).

### Alder Above- and Belowground Traits Vary Across Communities

Alder growing in different plant communities exhibited contrasting above- and belowground traits. Alder in shrublands were taller and had a greater basal area (**Table 2**). Shade leaves in alder shrublands also had a greater SLA, higher %N, and lower N_area_ than their counterparts growing in alder savannas (**Table 3**). Leaves throughout the canopy of alder growing in shrublands had higher %P and P_area_, a lower N:P, and a more depleted δ^15^N signature. The alder communities did not differ in leaf C:N or sun leaf %N, N_area_, or SLA. Alder litter collected within alder shrublands had a relatively high %N and N_area_, indicating a low N resorption efficiency (41.2%). In contrast, P resorption efficiency in alder shrublands was 62.4%.

**Table 2:** Structural traits of alder growing in two plant communities.

Community	Height (cm)	Basal Area Per Shrub (cm^2^ stem)	Basal Area (cm^2^/ m^2^ ground)	Nodule Biomass (g/ m^2^ ground)	Nodule Biomass (g/ cm^2^ stem)
Alder Shrubland	267.89 ± 8.13 *	391.94 ± 69.62 *	64.86 ± 16.65 *	18.54 ± 6.14 *	**0.47 ± 0.25**
Alder Savanna	111.55 ± 7.56 *	32.18 ± 7.38 *	11.39 ± 2.42 *	3.64 ± 1.21 *	**0.44 ± 0.15**

Averages are given ± standard error. Asterisk (*) and bold indicate statistically significant differences between communities (p < 0.05).

**Table 3:** Leaf traits of alder growing in two plant communities. Averages are given ± standard error.

Leaf Type	Community	SLA (cm^2^/ g)	δ^15^N	C:N	%N	N_area_ (g N/ m^2^ leaf)	N:P	%P	Parea (g P/ m^2^)
Sun	Alder Shrubland	120.55 ± 7.09	-1.74 ± 0.11 *	21.01 ± 1.73	2.33 ± 0.06	2.03 ± 0.08	14.40 ± 0.36 *	0.16 ± 0.01 *	0.14 ± 0.01 *
	Alder Savanna	116.56 ± 6.29	-1.36 ± 0.09 *	19.25 ± 0.88	2.25 ± 0.12	1.92 ± 0.09	20.43 ± 1.12 *	0.11 ± 0.01 *	0.10 ± 0.01 *
Shade	Alder Shrubland	160.6 ± 7.1 *	-1.76 ± 0.15 *	21.70 ± 1.76	2.29 ± 0.05 *	1.49 ± 0.07 *	14.67 ± 0.40 *	0.16 ± 0.01 *	0.10 ± 0.01 *
	Alder Savanna	120.42 ± 4.9 *	-1.22 ± 0.08 *	23.08 ± 0.85	1.98 ± 0.08 *	1.71 ± 0.10 *	23.52 ± 2.65 *	0.09 ± 0.01 *	0.07 ± 0.00 *
Litter	Alder Shrubland	151.16	-2.26 ± 0.18	32.61 ± 0.50	1.56 ± 0.02	1.03 ± 0.01	24.46 ± 2.76	0.07 ± 0.01	0.05 ± 0.01

Asterisk (*) and bold indicate statistically significant differences between communities (p < 0.05).

**Figure 2:**
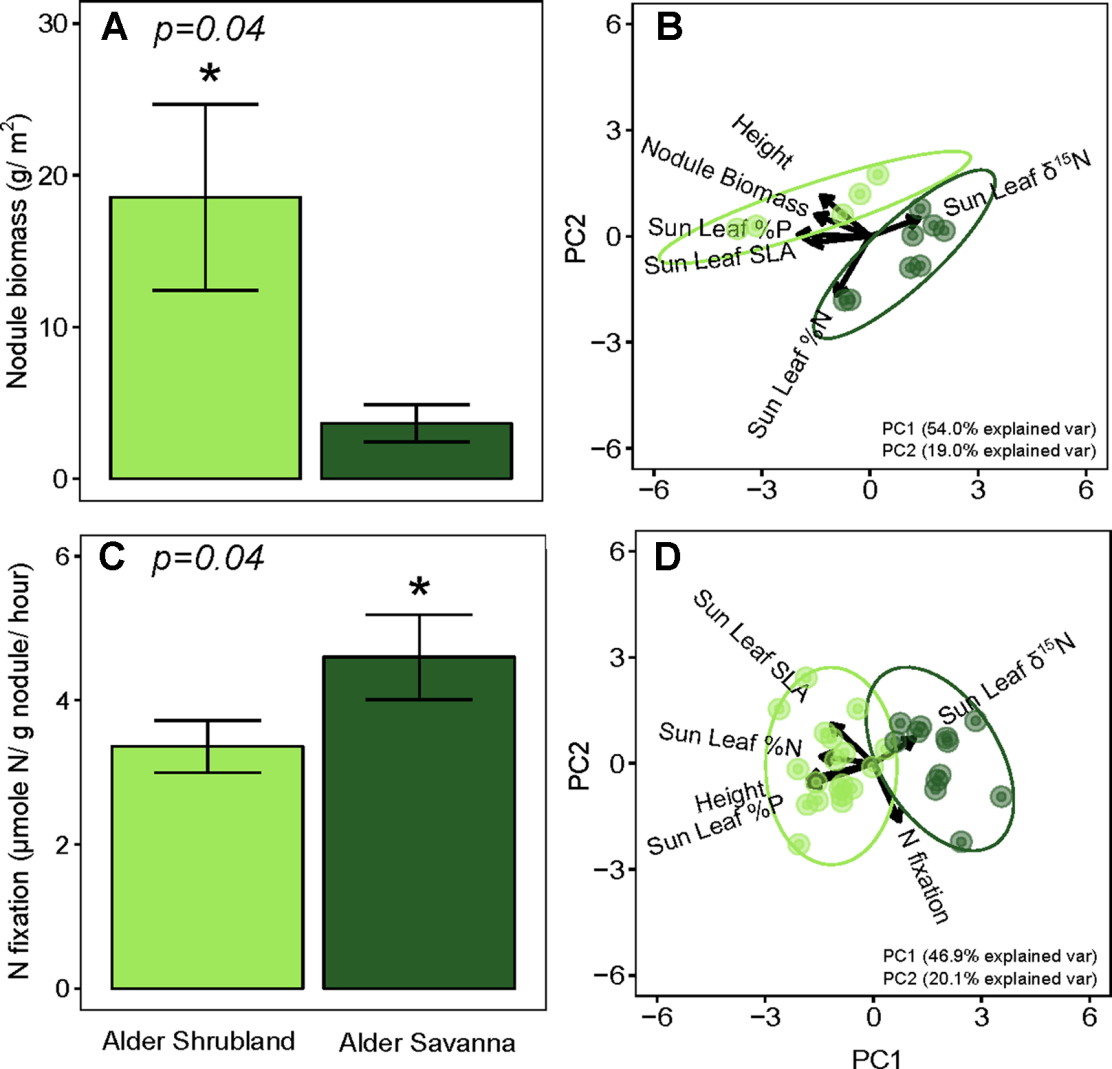
Below- versus aboveground traits of alder growing in shrublands and savannas. Alder nodule biomass **(A)** and its relationship to aboveground alder traits **(B)**, rates of N fixation **(C)** and its relationship to aboveground alder traits **(D)**. Asterisks (*) indicate statistically significant differences between communities (p < 0.05).

Nodule biomass per m^2^ ground was greater in alder shrublands than in alder savannas (**Table 2**, **Figure 2**, p = 0.04). When the nodule biomass was normalized to stem basal area, there was no significant difference between alder shrublands and alder savanna (**Table 2**). PCA of aboveground alder traits collected alongside nodule biomass showed a distinct separation of community traits along PC1, which explained 54.0% of the observed variation (**Figure 2B**, **Supplementary Table 2**). PC1 was positively correlated sun leaf δ^15^N and negatively correlated with nodule biomass, height, sun leaf SLA, sun leaf %N, and sun leaf %P.

N fixation rates within nodules were lower for alder growing in shrublands than alder growing in alder savannas (*P* = 0.04, **Figure 2C**). Similar to the pattern seen with nodule biomass, PCA of aboveground traits and N fixation exhibited a strong separation of communities (**Figure 2D**, **Supplementary Table 3**). PC1 explained 46.9% of variation in the dataset and was positively correlated with sun leaf δ^15^N and negatively correlated with N fixation rate, height, sun leaf SLA, sun leaf %N, and sun leaf %P.

**Figure 3:**
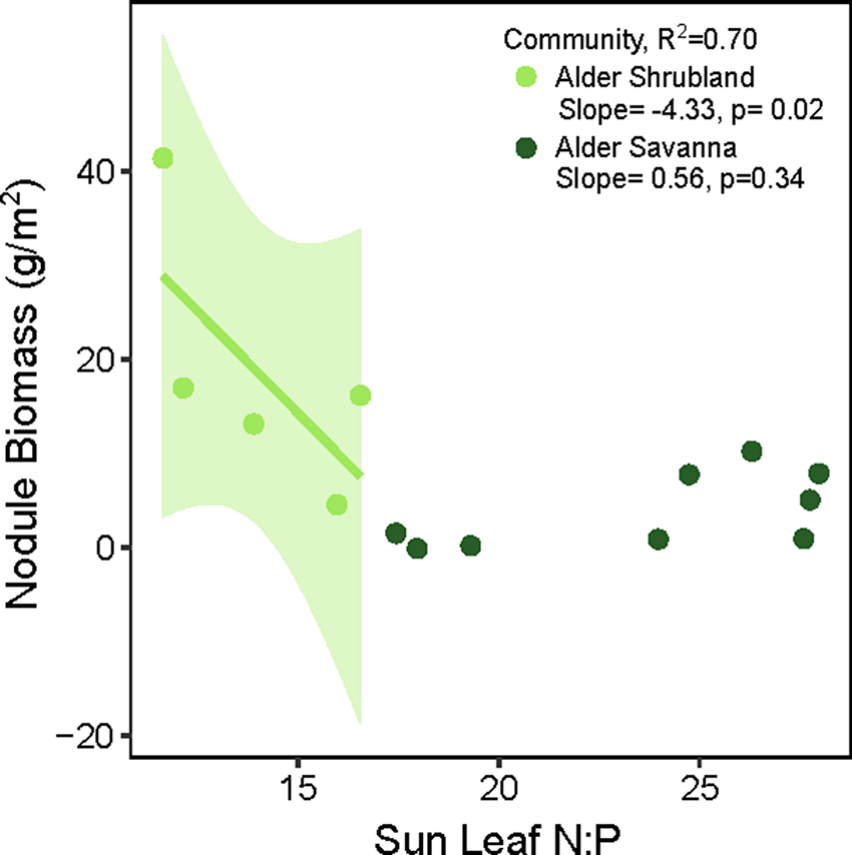
Alder nodule biomass in relation to sun leaf N:P (g:g basis). In alder shrubland nodule biomass plots, there were 4-8 alder individuals so the N:P ratio on the y axis is an average value. The colored line and shaded 95% confidence intervals denote significant relationship between nodule biomass and sun leaf N:P within the alder shrubland community.

The overall rate of N inputs via N fixation was primarily driven by variation in nodule biomass. Fixation rates were higher in nodules from alder savannas, but the higher nodule biomass within alder shrublands more than compensated for their lower nodule fixation rate. Alder shrublands had nodule biomass that was roughly four times that of alder savannas, but N fixation per unit nodule biomass in alder savannas was only 37% higher than rates in observed in shrublands. The greater nodule biomass within alder shrublands was associated with lower leaf N:P and there was a significant negative correlation between sun leaf N:P and nodule biomass in this community (**Figure 3**). Alder in savannas, however, had higher sun leaf N:P and no relationship between leaf N:P and nodule biomass.

### Peak Season and Annual Rates of N Fixation

Alder shrublands had an average peak season N fixation rate of 62 µmole N m^-2^ h^-1^ (±22 SE) while alder savannas had an averaged 17 µmole N m^-2^ h^-1^ (±6 SE). There was a wide range in N fixation rates per unit ground area within both communities, predominately driven by nodule biomass variation. When these peak season N fixation rates were integrated to yearly fluxes, annual N fixation by alder shrublands was over 3.5 times thats of alder savannas (**Table 4**).

**Table 4:** Annual N fixation within alder shrublands and alder savannas. Rates from this tundra ecosystem are at the lower end of the range reported for alder N fixation in boreal regions.

Study	Species	Biome/Habitat	Methods	Annual N fixation (g N/ m^2^/ yr)
This study	*A. viridis spp. fruticosa*	Tundra (Alder shrubland community)	_15_N_2_ incubation of nodules Soil core collection for nodule biomass	1.95 ± 0.68
This study	*A. viridis spp. fruticosa*	Tundra (Alder savanna community)	_15_N_2_ incubation of nodules Soil core collection for nodule biomass	0.53 ± 0.19
Mitchell and Ruess (2009)	*A. viridis spp. fruticosa*	Boreal (post-fire succession)	ARA incubation of nodules Soil core collection for nodule biomass	0.25 - 0.66
Uliassi and Ruess (2002)	*Alnus incana spp. tenuifolia*	Boreal (floodplain, unfertilized)	ARA incubation of nodules Soil core collection for nodule biomass	3.9 - 8.8
Ruess et al. (2009)	*Alnus incana spp. tenuifolia*	Boreal (alder stands impacted by canker)	_15_N_2_ incubation of nodules Soil core collection for nodule biomass	2.2 - 10.7
Ruess et al. (2013)	*Alnus incana spp. tenuifolia*	Boreal (floodplain, unfertilized)	_15_N_2_ incubation of nodules Soil core collection for nodule biomass	2.6 - 3.8
Crocker and Major (1955)	*Alnus viridis spp. crispa*	Boreal (recently deglaciated area)	Annual N increment in soil 40 year period	6.2
Van Cleve et al. (1971)	*Alnus incana spp. tenuifolia*	Boreal (floodplain, unfertilized)	Annual N increment in soil 20 year period	15.6

### Alder’s Impact on Local N Cycling

**Figure 4:**
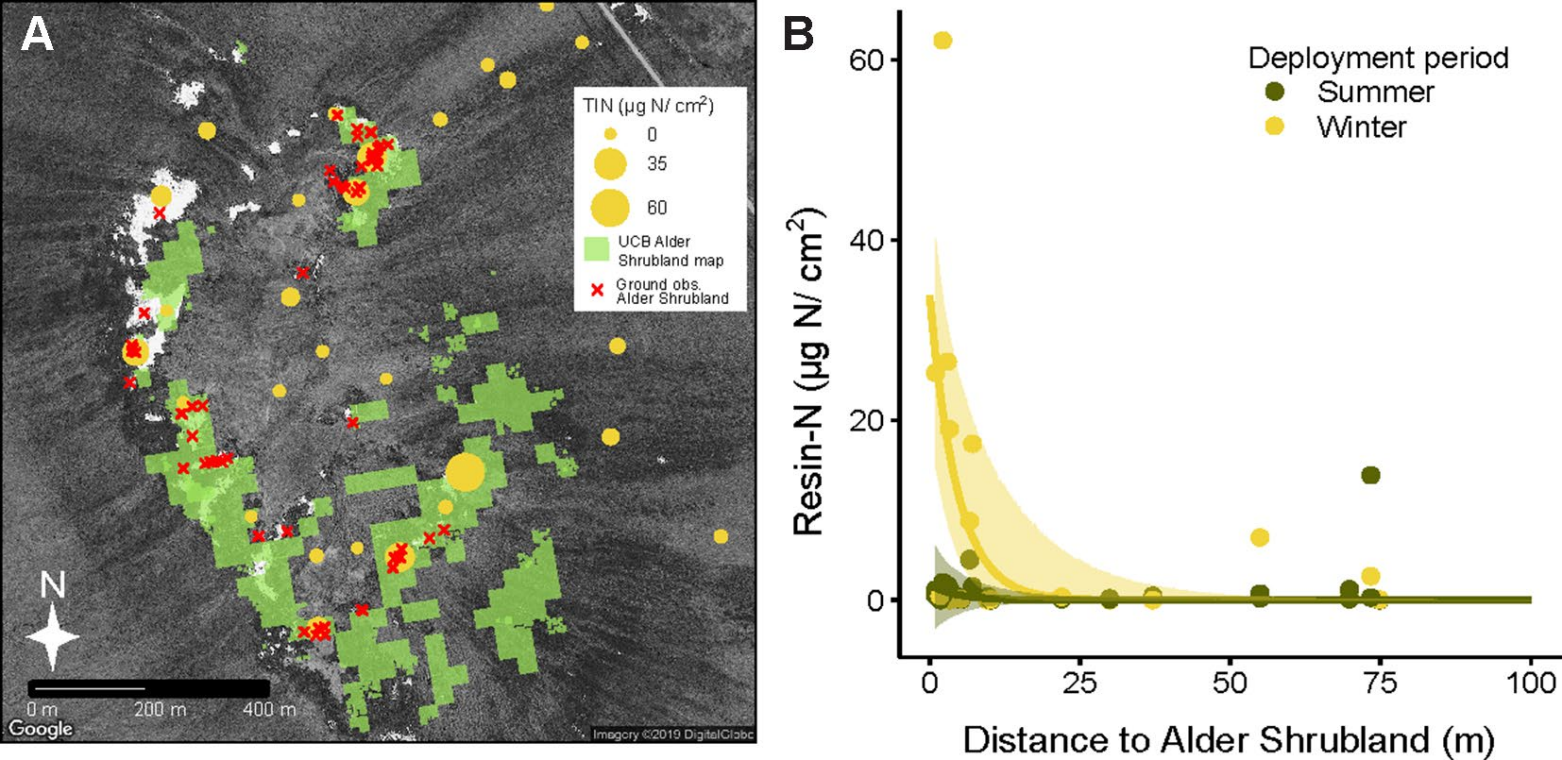
Inorganic N availability (Resin-N) at Kougarok Hillslope increases with proximity to alder shrublands. Light green areas in panel **A** represent an unsupervised classification-based (UCB) alder shrubland map derived from multisensor data. The accuracy of the alder shrubland map was verified with ground-based observations (red x’s). Resin-N was measured with ion-exchange resins deployed across the hillslope site and was found to decrease with increasing distance from alder shrublands (panel **B**). Shaded regions in panel B represent 95% confidence intervals fit to negative exponential model with separate intercepts for summer and winter seasons. Several near-zero resin-N values from 100 m to 380 m distance are not pictured in panel B but were included in model fitting.

Deployment period (winter vs summer) and plant community both had a significant impact on total inorganic nitrogen extracted from resin capsules (Resin-N, **Supplementary Figure 2A**, *P* = 0.003 and *P* = 0.013 respectively). Overall, resin-N in winter was greater than resin-N in summer, and alder shrubland communities tended to have the highest levels of resin-N. Within the each of the six communities at Kougarok Hillslope, however, there was a high degree of variation in resin-N replicates. P availability followed a similar pattern but with greater variation and only the deployment term was significant in the model (**Supplementary Figure 2B**, *p* = 0.019). The extreme variation in nutrient availability within the six plant communities led us to hypothesize that high resin-N was associated with proximity to alder shrublands rather than a location’s community classification. Comparing the modern UCB alder shrubland map (**Figure 4A**) with locations of resins confirmed that resins near alder shrublands were indeed higher in resin-N (**Figure 4B**). The negative exponential model was best fit with a deployment-specific intercept term (A) and a common extinction coefficient (b = 0.22, 95% CI from 0.09 to 0.36). The intercept term for resins deployed in winter, A_winter_ was higher (33.84, 95% CI from 19.20 to 48.49) than that for resins deployed in the summer (A_summer_, 1.73, 95% CI from -3.71 to 7.17).

**Figure 5:**
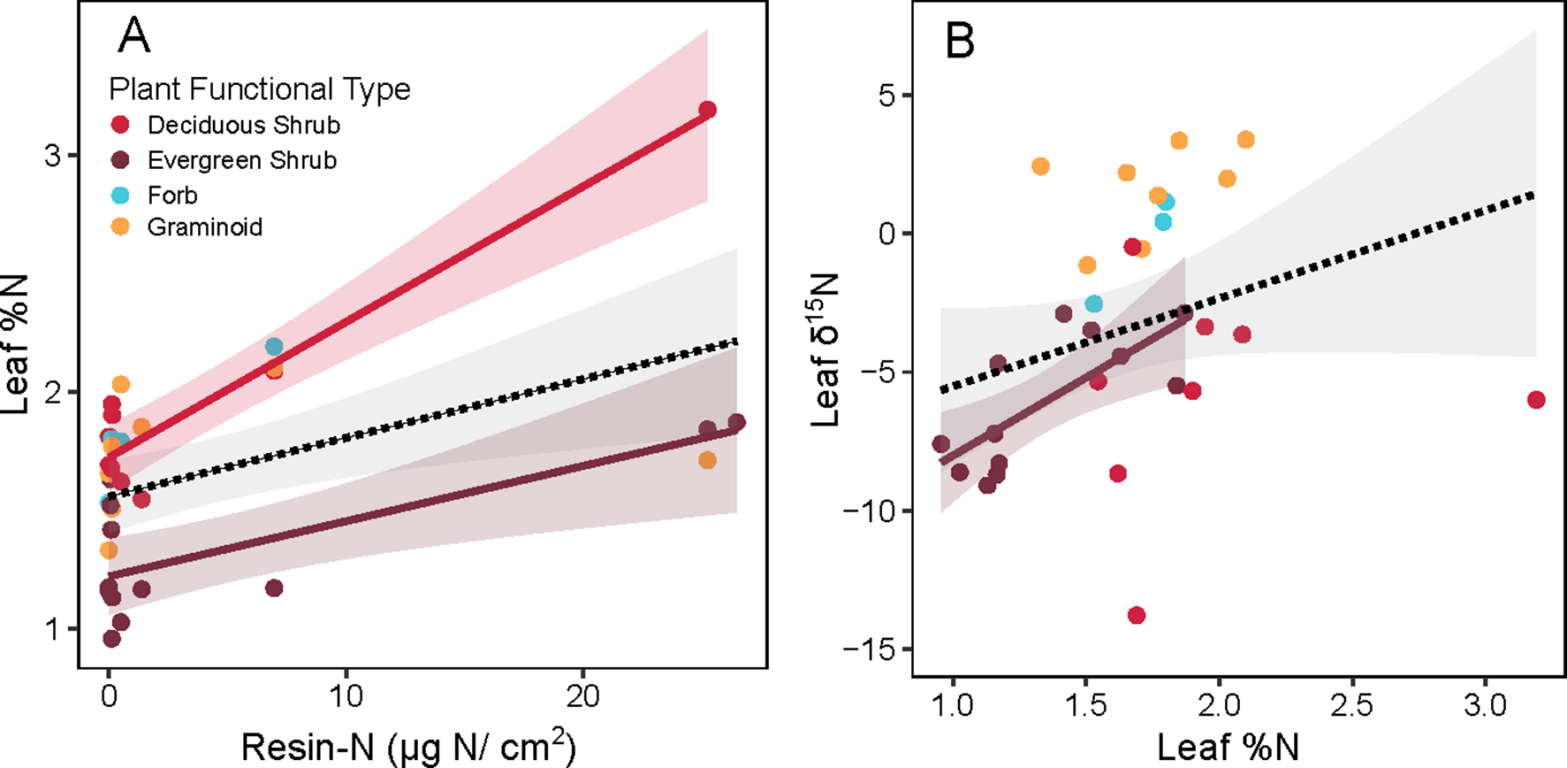
Relationship between inorganic N availability (Resin-N) and leaf chemistry of non-alder plant species at Kougarok Hillslope. Panel **A** shows Resin-N versus leaf %N while panel **B** shows Leaf %N versus Leaf %15N. Colored lines and shaded 95% confidence intervals denote significant slope of a given plant functional type (p < 0.05). Dashed lines and grey 95% confidence intervals represents a significant slope fit across all PFTs (p < 0.05).

When leaf %N of non-alder species was examined, overall leaf %N increased with resin-N (**Figure 5A**, overall slope = 0.002, *p* = 0.004). Overall, this pattern was weak, however, and was driven primarily by the few locations where resin-N was high. Within PFTs, this trend was significant for both deciduous (slope = 0.005, *p* < 0.001) and evergreen shrubs (slope = 0.002, *p* < 0.002), but the leaf %N of forbs and graminoids was not significantly correlated with resin-N. The δ^15^N signature of non-alder leaves tended to be heavier when leaves had higher leaf %N (**Figure 5B**, overall slope = 6.4, *p* < 0.001). Within PFTs, however, this slope was only significant for evergreen shrubs (slope = 4.32, *p* < 0.001).

### Extent of Alder Shrublands Through Time

**Figure 6:**
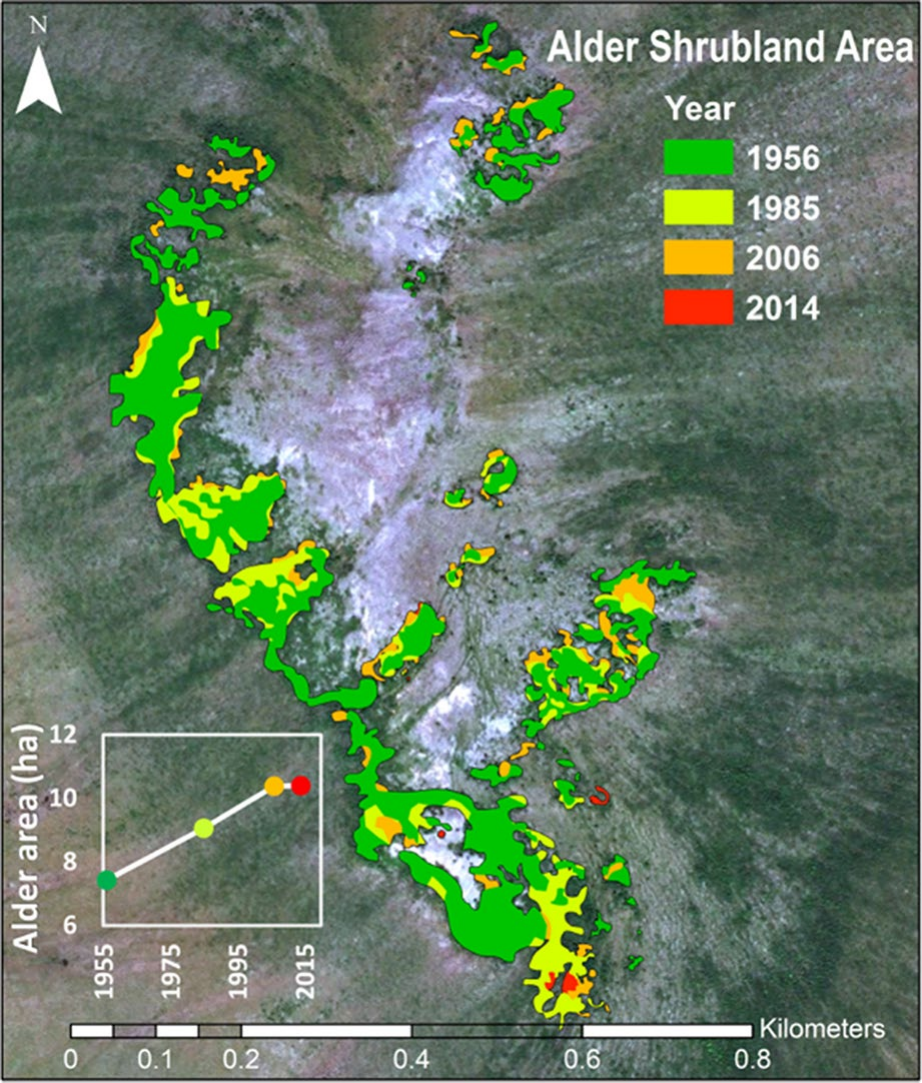
Expansion of alder shrubland at the Kougarok Hillslope (1956-2014). The photo-interpreted area alder shrublands through time are overlaid on the 2014 WorldView2 image of the site.

Time-series image analysis showed that alder shrublands at Kougarok Hillslope have expanded their range by 40% since 1956 (**Figure 6**, **Table 5**). This analysis does not include alder in savannas, which could not be differentiated from other deciduous shrubs given their low density and short stature in these areas. We found alder shrubland cover increased continually from 7.4 ha in 1956 to 10.4 ha in 2014. Rates peaked between 1956 and 1985 and alder shrubland expansion appeared to slow in the 2000’s (inset in **Figure 6**). The average rate of alder shrubland expansion was 513 m^2^ year^-1^ and most of this occurred by infilling gaps between patches of existing alder shrublands.

**Table 5:** Alder shrubland expansion at Kougarok Hillslope since 1956.

Year	Cover by Alder Shrublands
1956	74,231 m_2_
1985	90,621 m_2_
2006	103,812 m_2_
2014	103,967 m_2_
1956-2014	29735 m_2_ increase
Rate of change	513 m_2_/ year
Percent change (1956-2014)	40%

## Discussion

We quantified N fixation by alder across the Kougarok Hillslope to better understand the role alder may play in warming tundra ecosystems. We hypothesized that the tall, dense alder in shrublands would have a greater impact on local N cycling than the short, dispersed alder in savanna communities and our findings support this hypothesis. Annual rates of N fixation within alder shrublands were over three times as high as N fixation in alder savannas, and N-limited tundra plant species growing in communities adjacent to alder shrublands had greater access to N. The observed expansion of alder shrublands therefore has important implications for N cycling within tundra ecosystems, and our data provide intriguing evidence that we may be able to predict the biogeochemical consequences of alder expansion across the tundra based upon aboveground alder traits.

### Alder Shrublands Have an “Open” N-Cycle That Impacts Neighboring Plants and Soils

Context for the N fixation rates presented in this study can be provided by comparison with the N required for annual net primary productivity (N_req_). We estimate the N_req_ of the entire alder shrubland community to be 13 g N m^-2^ year^-1^ and the N_req_ of the entire alder savanna community to be 11 g N m^-2^ year^-1^ based on data from biomass harvests of understory species, shrub allometries, and gapfilled tissue %N and rhizome pools based on existing arctic literature (Salmon et al., in preparation). Arctic plant communities lacking alder have N_req_ ranging from 0.42-4.40 g N m^-2^ year^-1^ (Shaver and Chapin, 1991). The annual N fixation rates reported in this study (0.53-1.95 g N m^-2^ year^-1^) are at the lower end of the range of reported values for alder growing in boreal regions though reported rates vary widely between habitat types as well as methodologies (**Table 4**). The lower rates of N inputs by tundra versus boreal alders could be the result of a colder climate, lower phosphorus availability, or a shorter growing season. However, when we consider these N inputs by tundra alders next to the N_req_ of neighboring tundra communities, their magnitude is striking. Within alder shrubland and alder savanna communities, N fixation supplies 16% and 5% of N_req_ respectively. Typically, plants with a greater reliance on symbiotic N fixation are associated with leaf δ^15^N near zero (Shearer and Kohl, 1986), but we found alder leaf δ^15^N in shrubland communities was more negative than alder leaf δ^15^N in savanna communities (**Table 3**). This does not discredit the higher proportion of N_req_ met by N fixation in the alder shrubland community we calculate, however. Increased N contribution from ectomycorrhizal symbionts or greater reliance on organic N forms in alder shrublands could similarly explain the depleted δ^15^N signature of alder shrublands (Hobbie and Hobbie, 2006; Yano et al., 2009).

The high proportion of N_req_ met by N fixation in alder shrublands in conjunction with the observed increased resin-N in soils located near alder shrublands soils suggest that alder shrublands do not have a very tightly constrained N cycle. Alder shrublands may fail to retain N due to their high rates of litter N deposition and their location in well-drained uplands where they are presumably susceptible to downhill losses of N in the form of litter and leachate. We found that alder leaf litter from shrublands was high in N (1.56% N by weight). N resorption efficiency was still higher than reported for boreal alders (41.2% versus 13.9%; Uliassi and Ruess, 2002). The N resorption efficiency of tundra alders we observed, however, was lower than the 65% figure cited for non-fixing tundra species (Jonasson and Shaver, 1999). By applying allometric equations relating basal area to aboveground biomass and assuming leaves makes up roughly 80% of new growth, we can calculate the annual flux of N in alder leaf litter to be around 3.8 g N m^-2^ (Berner et al., 2015; personal communication L. Berner). The downslope movement of alder litter and leachate is therefore a likely mechanism behind the elevated levels of resin-N we observed within a 20 m radius of alder shrublands. Resin-N was greatest when resins were deployed through the winter season, potentially due to winter decomposition of alder litter (Kielland et al., 2006; Buckeridge et al., 2010), moisture limitation of summer N mineralization, or competition between resins and roots for N during the growing season. Transport of N from alder shrublands to surrounding soils and water bodies could therefore have important implications for biogeochemical cycling across arctic landscapes. Related work at the Kougarok hillslope suggests that nitrate from these alder shrublands is transported downslope during rainfall events (McCaully et al., in preparation,). Future research on the hydrological transport of alder-sourced N across arctic landscapes is needed and we believe that an expanded version of our UCB alder shrubland map could prove integral to these efforts.

The positive relationship between resin-N and leaf %N of deciduous and evergreen shrubs suggests that the hotspots in resin-N associated alder shrublands (**Figure 4B**) explained some of the observed variation in shrub leaf %N. This suggesting that evergreen and deciduous shrubs may have access to N “leaked” from alder shrublands. With the data at hand it is not possible to directly attribute this increased leaf %N to increased reliance on alder-fixed N (see Binkley et al., 1985). Leaf δ^15^N signatures tended to less depleted in when leaf %N was high. This could indicate an increased dependence on recently-fixed N since δ^15^N signatures close to zero are associated with N fixation (Shearer and Kohl, 1986). However, alder shrubland leaf δ^15^N was around -1.75 (**Table 3**), so this source is unlikely to explain the high degree of depletion we observed in evergreen and deciduous shrubs (around -5, **Figure 5B**). An altered balance between fractionating processes in the N cycle may explain the low values observed in **Figure 5B**. At the Kougarok Hillslope, graminoid and forb δ^15^N signatures were near or greater than zero as is common for non-mycorrhizal plants. These PFTs are known to access N from deeper mineral soils (Hewitt et al., 2019) which may buffer them from the isotopic signature of alder-derived N as well as explain why %N of these PTFs was not correlated with inorganic N availability of surface soils.

### Alder Shrublands Are Expanding

The observed expansion of alder shrublands at Kougarok Hillslope has important implications for N inputs at this site. If the observed rate of N fixation by alder shrublands is applied to annual area of alder shrubland from 1956-2014, we see that the 40% increase in alder shrubland area was associated with a 22% increase in N fixation. Such an increase in available N has the potential to reshape plant productivity, alter plant community composition, and accelerate microbial decomposition in downslope, N-limited communities (Shaver et al., 2001; Mack et al., 2004). Nitrogen saturation also has the potential to increase N leaching and impact downstream ecosystems (Hiltbrunner et al., 2014). Expansion of alder shrubland communities at Kougarok Hillslope was associated with colonization of rocky, well-drained outcroppings near the crest of the hill and did not extend down into the tundra lowlands. This heterogeneous pattern of expansion parallels observations made farther north in Alaska (Tape et al., 2012). The 513 m^2^ year^-1^ of alder expansion reported here, therefore, could be used along with topographical maps and classification of suitable alder shrubland terrain to estimate future rates of alder expansion in Alaska and Northwest Canada. Care should be taken not to apply this rate to the entire arctic biome.

### Sources of Variation in Alder N Fixation Rates

We posit that the variation in annual N fixation by alder shrubland and alder savanna communities was driven by a combination of P limitation and soil moisture dynamics. Nodule biomass exerted stronger control on annual N fixation inputs than the rates of fixation within individual nodules. The relationship between leaf N:P and nodule biomass suggests P availability varies across communities and higher sun leaf N:P was associated with lower nodule biomass. Alder savanna sun leaves generally had N:P over 20, a threshold often used to signify P limitation (Güsewell, 2004; Koerselman and Meuleman, 1996). Fertilization studies in tussock tundra plant communities have shown them to be jointly limited by N and P availability (Chapin and Shaver, 1985) and the regular spacing of alder growing in graminoid dominated tundra has been attributed to intra-specific competition for nutrients (Chapin et al., 1989). Furthermore, P availability was found to limit alder nodule production in a primary succession within a boreal floodplain (Uliassi and Ruess, 2002). The low availability of soil P and the higher foliar N:P in alder savannas, the propensity for graminoid-dominated tundra to be jointly N and P limited, and the fact that alder access to P limits alder nodule biomass in boreal ecosystems together suggest our observed differences in nodule biomass at Kougarok Hillslope are driven in part by P limitation within alder savanna communities.

Differences in soil moisture between the two plant communities may also be a key factor driving the observed variation in N fixation by alder shrubland and savanna communities. Though the surface soil moisture within the two plant communities was similar, the overall soil profiles differed greatly in their drainage potential and alder savannas had higher soil moisture deeper in the soil profile. The shallow, sloping soils within alder shrublands were underlain by bedrock and would likely drain well during the growing season while the deep, flat soils in alder savannas would tend to retain moisture were are often saturated beneath the soil surface. The wet conditions in alder savannas were likely also a result of the underlying permafrost in these soil profiles: this impermeable layer would force the water table to remain perched near the soil surface. Though root nodules create an anaerobic zone for N fixation by *Frankia* bacteria, alder roots themselves do not appear to thrive in saturated soils (Tape et al., 2012). If the rooting zone of alder in savanna communities is restricted to the dry, organic horizon, alder access to nutrients in mineral horizons could be reduced. This could limit alder biomass production as well as slow the C supply to N-fixing symbionts.

### Scaling Up Alder N Fixation

The strong association between alder basal area and rates of N fixation we observed across alder shrubland and alder savanna communities suggests that aboveground biomass could be a reliable scaler for estimating rates of N fixation by alder. Nodule biomass of alder shrublands was greater than in alder savannas when nodule biomass was expressed per unit ground area but nodule biomass of the two communities was similar when expressed per unit alder stem basal area (**Table 3**). The range of nodule biomass per m^2^ ground was similar to that reported for the same species in boreal regions (Mitchell and Ruess, 2009) and N fixation rates within nodules were on the lower end of the range reported for boreal *A. incana* ssp. *tenuifolia* and *A. viridis* ssp. *crispa* measured using the same ^15^N_2_ method (Anderson et al., 2004; Ruess et al., 2013). Work by Rhoades et al (2001) and Uliassi and Ruess (2002) in the boreal zone found similar positive correlations between N indices (resin-N, N fixation) and alder aboveground biomass (height, LAI). These lines of evidence suggest the relationship between alder aboveground biomass and inputs of N via symbiotic fixation may be consistent across boreal and arctic ecosystems and therefore may be an integral part of quantifying landscape level fluxes of N in high latitudes. These findings substantiate the assumptions in Epstein et al. (2000) and Menge et al. (2009), both of which model N fixation of alders by scaling this flux to plant biomass. Scaling N fixation rates from this study to other tundra landscapes, however, should be undertaken with caution. There are known sources of variation not considered in this study: nitrogen fixation by boreal alder is impacted by choice of *Frankia* bacterial partner (Anderson et al., 2009; Ruess et al., 2013), fungal infection (Ruess et al., 2009; Nossov et al., 2011), and stand age (Mitchell and Ruess, 2009). Further research is needed to quantify the impact these factors have on tundra alders.

### Implications for Modeling Warming Arctic Ecosystems

Here we have shown that alder can be an important source of N to surrounding tundra plant communities and that N-fixation rates vary the community and landscape position the alder occupies. The area of tall alder shrublands has increased over past decades in upland areas and may increase further as tundra regions continue to warm. This expansion has important consequences for tundra N cycling, especially in areas downslope of alder shrublands. Our data both inform the mechanistic representation of N-fixation in models and provide observations to evaluate model projections.

Though alder has long been present in tundra ecosystems, few large-scale, high-latitude models include an N-fixing PFT. Epstein and colleagues (2000) incorporated *Alnus* species in ArcVeg, but other arctic models do not (ie, Rupp et al., 2000; Euskirchen et al., 2009). Climate-scale earth system models not simulate N-fixing species in high latitudes. The data reported in this study provide useful constraints for initiating implementation of a N-fixing shrub PFT in large-scale models. Our results suggest that N fixation by alder could be scaled to the landscape level using aboveground alder traits (e.g. height and biomass) that can be derived from remote-sensing products. The observed seasonal variation in resin-N may also help constrain alternative hypotheses for the processes driving plant nutrient uptake, and the seasonal timing of uptake (Riley et al., 2018). Capturing the variation in N fixation observed in this study may require community specific- parameterization of a N-fixing PFT or dynamic trait distributions that respond to environmental cues (Wullschleger et al., 2014). Parameterization of an N-fixing PFT would likely help capture changing vegetation dynamics of arctic ecosystems and could lead to more robust predictions of C and N feedbacks at high latitudes.

## Data Availability Statement

All data are publicly available. DOIs and hyperlinks are provided in the methods section.

## Author Contributions

VS led data collection in field and laboratory and analyzed data with significant assistance from CI. AB classified vegetation communities and plant species at the site, established plots, and performed field work. JK performed remote sensing neural network analysis and helped with field measurements. ML analyzed historic images from the site and helped with field measurements. PT and SW helped with field measurements and gave feedback on early drafts of the analysis. VS and CI wrote the first draft of the manuscript and all authors contributed to the final draft.

## Funding

This manuscript has been authored (in part) by UT-Battelle, LLC, under contract DE-AC05-00OR22725 with the US Department of Energy (DOE). The US government retains and the publisher, by accepting the article for publication, acknowledges that the US government retains a nonexclusive, paid-up, irrevocable, worldwide license to publish or reproduce the published form of this manuscript, or allow others to do so, for US government purposes. DOE will provide public access to these results of federally sponsored research in accordance with the DOE Public Access Plan (http://energy.gov/downloads/doe-public-access-plan).

## Conflict of Interest

The authors declare that the research was conducted in the absence of any commercial or financial relationships that could be construed as a potential conflict of interest.
