# Supplementary material for: Alder Distribution and Expansion Across a Tundra Hillslope: Implications for Local N Cycling
Source: Front Plant Sci. 2019 Oct 16;10:1099. doi: 10.3389/fpls.2019.01099 (PMC6807776; doi:10.3389/fpls.2019.01099)
Supplement: Supplementary file 1 [file DataSheet_1.pdf]

**SUPPLEMENTAL MATERIALS**

**Title:** Alder distribution and expansion across a tundra hillslope: implications for local N cycling

**Supplementary Figure 1**. Spectral properties of Alder shrubland plots, based on EO-1 Hyperion. Back lines represent spectra from alder shrubland plots while grey lines represent spectra from other plant communities present at Kougarok.

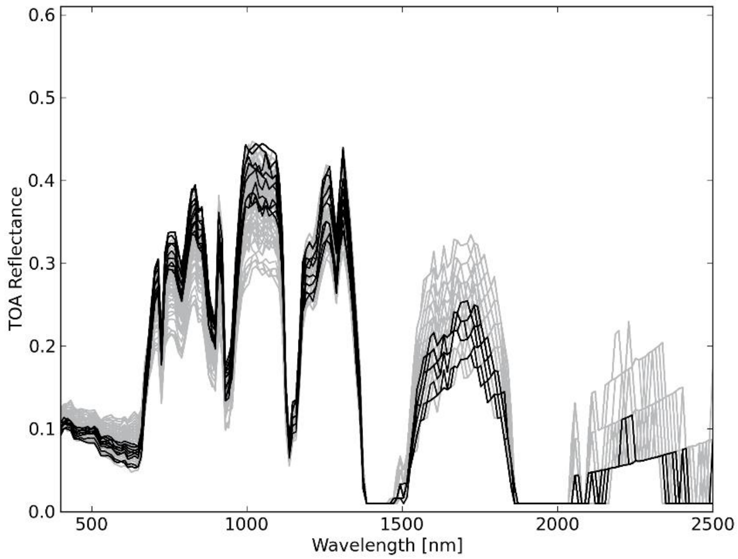


*Notice: This manuscript has been authored by UT-Battelle, LLC under Contract No. DE-AC05-00OR22725 with the U.S. Department of Energy. The United States Government retains and the publisher, by accepting the article for publication, acknowledges that the United States Government retains a non-exclusive, paid-up, irrevocable, world-wide license to publish or reproduce the published form of this manuscript, or allow others to do so, for United States Government purposes. The Department of Energy will provide public access to these results of federally sponsored research in accordance with the DOE Public Access Plan (http://energy.gov/downloads/doe-public-access-plan).*

**Supplementary Figure 2**. Resin-N (Total inorganic N extracted from resins, panel a) and Resin-P (panel b) availability. Community type, deployment date, and an interaction between community type and deployment date all had a significant effect on Resin-N ($p < 0.02$). Deployment data had a significant effect on resin-P ($p = 0.02$).

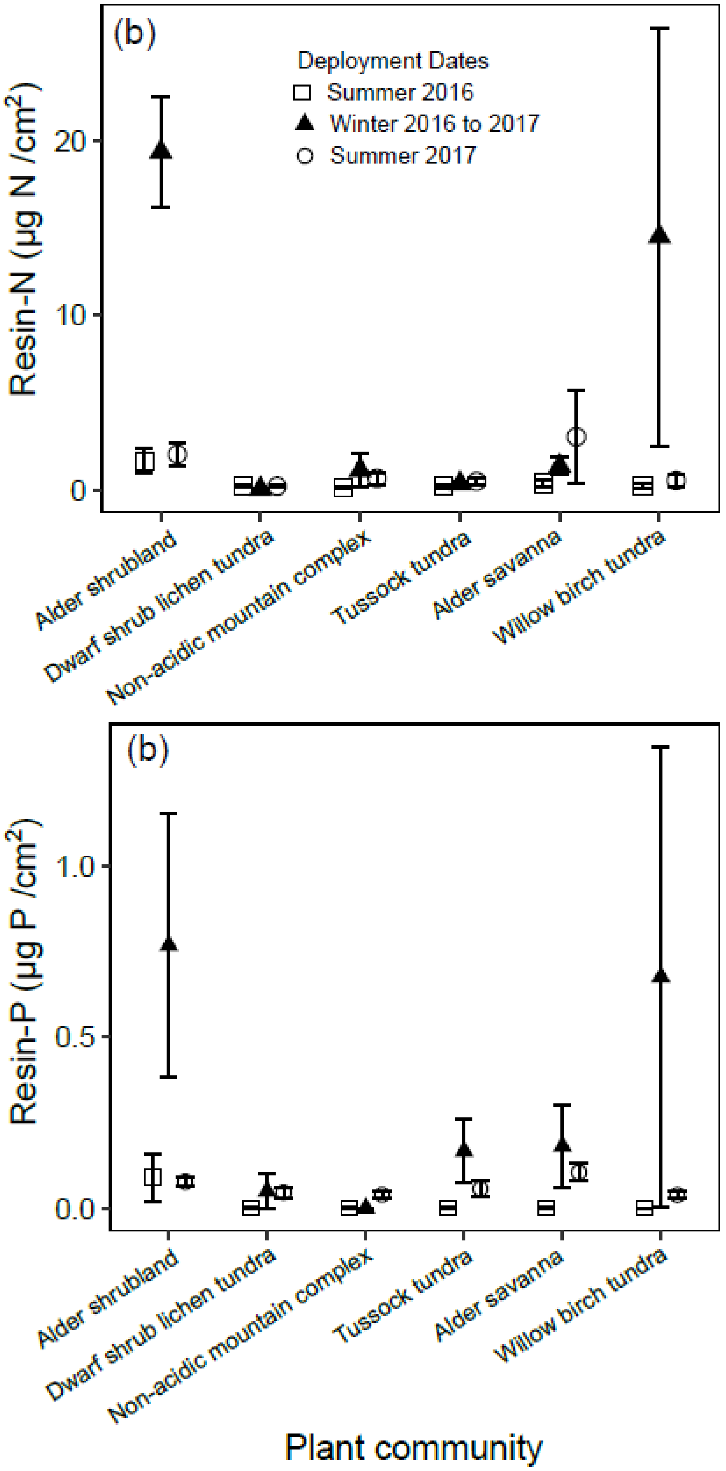

**Supplementary Table 1**. Spectral and topographic remote sensing data used in the classification of Alder shrubland. (DN: Digital Numbers)

| Sensor | Variable | Unit | Collection Date | Resolution | # of bands |
|---|---|---|---|---|---|
| SPOT-5 | Green, Red, NIR (500-900 nm) | DN | June-September 2009-2012 | 2.5 m | 3 |
| USGS IfSAR DEM | Elevation | m | July 2012 | 5 m | 1 |
| EO-1 | 198 spectral bands (400-2500 nm) | DN | June 24, 2015 | 30 m | 198 |
| Landsat 8 | 9 spectral bands (400-2290 nm) | DN | August 17, 2016 | 30 m | 9 |

**Supplementary Table 2**. Principal Components analysis of Alder nodule Biomass and aboveground traits. Asterisk (*) and bold type denote variable had a significant impact on Principal component. Significance was determined by bootstrapping of eigenvectors (p <0.05).

| | *Eigenvectors* | | | | | |
|---|---|---|---|---|---|---|
| | **PC1** | **PC2** | **PC3** | **PC4** | **PC5** | **PC6** |
| Nodule Biomass (g/m$^2$) | -0.41 | 0.28 | -0.5 | -0.5 | 0.5 | -0.2 |
| Height (cm) | **-0.37 *** | 0.53 | 0.3 | 0.6 | 0.2 | -0.2 |
| Sun Leaf SLA (cm$^2$/g) | **-0.45 *** | -0.08 | 0.5 | -0.4 | -0.5 | -0.4 |
| Sun Leaf %N | **-0.26 *** | -0.76 | 0.2 | 0.2 | 0.5 | -0.1 |
| Sun Leaf $\delta^{15}$N | **0.37 *** | 0.23 | 0.7 | -0.4 | 0.5 | 0.1 |
| Sun Leaf %P | **-0.54 *** | 0.02 | 0.1 | -0.1 | -0.1 | 0.8 |

| | *Importance of Components* | | | | | |
|---|---|---|---|---|---|---|
| | **PC1** | **PC2** | **PC3** | **PC4** | **PC5** | **PC6** |
| Standard deviation | 1.80 | 1.07 | 0.9 | 0.6 | 0.6 | 0.2 |
| Proportion of Variance | 0.54 | 0.19 | 0.1 | 0.1 | 0.1 | 0 |
| Cumulative Proportion | 0.54 | 0.73 | 0.9 | 0.9 | 1 | 1 |

**Supplementary Table 3**. Principal Components analysis of Alder N fixation and aboveground traits. Asterisk (*) and bold type denote variable had a significant impact on Principal component. Significance was determined by bootstrapping of eigenvectors (p <0.05).

*Eigenvectors*

| | PC1 | PC2 | PC3 | PC4 | PC5 | PC6 |
|---|---|---|---|---|---|---|
| N fixation (µmole N/g nodule/ hour) | 0.21 | **-0.73 *** | 0.3 | -0.1 | -0.6 | -0.1 |
| Height (cm) | **-0.53 *** | -0.20 | 0.3 | 0 | 0.4 | **-0.68 *** |
| Sun Leaf SLA ($cm^2/g$) | **-0.34 *** | **0.50 *** | 0.4 | 0.4 | -0.6 | -0.1 |
| Sun Leaf %N | **-0.42 *** | 0.09 | -0.4 | -0.7 | -0.4 | 0 |
| Sun Leaf $\delta^{15}N$ | **0.33 *** | 0.32 | 0.6 | -0.6 | 0.2 | 0 |
| Sun Leaf %P | **-0.52 *** | -0.28 | 0.30 | 0 | 0.20 | 0.7 |

*Importance of Components*

| | PC1 | PC2 | PC3 | PC4 | PC5 | PC6 |
|---|---|---|---|---|---|---|
| Standard deviation | 1.68 | 1.10 | 0.9 | 0.8 | 0.7 | 0.3 |
| Proportion of Variance | 0.47 | 0.20 | 0.2 | 0.1 | 0.1 | 0 |
| Cumulative Proportion | 0.47 | 0.67 | 0.8 | 0.9 | 1 | 1 |
